# Examining the influence of deterrent and enhancement factors on QR-code mobile payment continuance intention: insights from PLS-SEM and IPMA analysis

**DOI:** 10.3389/fdata.2025.1679897

**Published:** 2026-01-22

**Authors:** Ashikur Rahman, Fahmid al Farid, Mohammad Abul Bashar, Jia Uddin, Arif Mahmud, Hezerul Abdul Karim

**Affiliations:** 1 Department of Information Technology and Management, Daffodil International University, Dhaka, Bangladesh; 2 Centre for Image and Vision Computing (CIVC), COE for Artificial Intelligence, Faculty of Artificial Intelligence and Engineering (FAIE), Multimedia University, Cyberjaya, Malaysia; 3 Department of Management, Faculty of Business Administration, University of Chittagong, Chittagong, Bangladesh; 4 AI and Big Data Department, Endicott College, Woosong University, Daejeon, Republic of Korea; 5 Department of Computer Science and Engineering, Daffodil International University, Dhaka, Bangladesh

**Keywords:** continuance intention, mobile financial service, PLS-SEM, QR-code mobile payment, UTAUT2, emerging economies

## Abstract

**Introduction:**

The rise of contactless payment has made quick response (QR) code-mobile payment (QR-MP) platform increasingly popular among mobile financial service (MFS) users, especially in emerging economies. It has been demonstrated that the ongoing use of QR payments can significantly drive the growth of emerging economies. However, despite its importance, the continued use of this technology has not been satisfactory. Thus, this study seeks to explore the modified Unified Theory of Acceptance and Use of Technology 2 (UTAUT2) model, including four additional constructs: amotivation (AM), alternative attractiveness (AA), QR transaction anxiety (QTA), and transaction convenience (TC) to examine the MFS users' sustained usage of QR payment.

**Methods:**

Data were collected from 247 MFS users in Bangladesh using an online survey and analyzed through SEM-PLS and non-linear analysis of IPMA.

**Results:**

The research findings reveal that effort expectancy is the most influential factor, and that both moderator factors, QTA and TC, are significant. However, social influence and hedonic motivation were found to be insignificant. Furthermore, our extended research model explains 76.5% of the variance in CINT without the moderation effect.

**Discussion:**

The IPMA findings help to find the best-performing variables and provide practical insights for this study. Theoretical and managerial implications are provided to enrich the existing literature on the study of information technology, indicating how MFS providers in developing countries can retain their existing users.

## 1 Introduction

The emergence of the QR payment system on a mobile phone in the present day is the consequence of the rapid evolution of mobile technology, which motivates users to make contactless payments using MFS applications (Gao et al., 2018). With its growing popularity and rapid adoption, it is anticipated that, worldwide QR-MP transaction volumes will reach $2.71 trillion by 2030, and the highest acceleration is observed in the growing economy (Statista, 2021). A decade ago, QR payment platforms were unreachable, particularly in the least developed countries. Yet, today, it has become an indispensable part of daily financial transactions, dominating not only in urban areas but also in rural settings (Shunmugasundaram et al., 2025; Azizah and Bintoro, 2023).

The expansion of cashless transaction platforms in Bangladesh has been rapidly driven by the advent of mobile financial services (MFS) in 2011. The integration of QR technology in cashless transactions has led to a significant growth of 1,200% between 2013 and 2021 (Rahgir, 2021), which have significantly expanded the MFS industry, with 20 million daily transactions amounting to over $400 million (Khurshid and Basher, 2023). The largest mobile wallet operator “bKash” has already deployed more than half a million QRs (Uddin, 2023). Being a fast-moving country, it has significant growth opportunities in its digital financial services and mobile payments landscape. Further, it has been also reported that the sustained usage of QR payment system leads to an increase in the gross domestic product (GDP) by 1.7% (Talukder, 2023). Bangladesh government is relentlessly working toward achieving the sustainable development goals (SDGs) by 2030 and toward becoming a digital Bangladesh (Monir et al., 2025; Bhattacherjee, 2001). To achieve this goal, the central bank launched Bangla QR, a uniform digital payment system aimed to significantly cut cash-based retail transactions and also lifted the limit on transactions done with Bangla QR codes, for which the users are not currently charged any transaction fees (Rahman et al., 2024).

Even with government support and major progress in digital payments, cash transactions have still dominated over the past five years. The Central Bank's report reveals that as of 2023, there are 71 million registered MFS accounts, of which only 32.3 million are only active, which indicates that a significant portion of users adopt initially for these services but do not continue using them regularly. Accordingly, several underlying factors may contribute to this issue. For instance, users may register for the service but engage with it only occasionally, resulting in a high number of dormant accounts (Islam and Mia, 2024). Additionally, some users may prefer alternative payment methods, such as cash or traditional banking (Nandru et al., 2024). Similarly, transaction anxiety could further deter users from maintaining active usage (Rusydi et al., 2024). In contrast, high transaction convenience ensures users satisfaction toward digital payment (Yogi and Pramudana, 2021). User's post-use amotivation may arise after a certain technology has been adopted, and consequently it causes discontinuation of the existing technology (Altrichter and Benoit, 2025). Although the above-mentioned factors are prioritized by many researchers, they are rarely examined simultaneously with UTAUT-2 model in the context of QR payment study. Therefore, it emphasizes the importance of investigating the determinants of continuance usage intention of QR payment system.

Prior empirical evidence confirm that the success of any information system depends on its continuance usage and not on its initial adoption (Bhattacherjee, 2001). While QR-payment research largely examined users' pre-adoption and usage behavior rather than users' post adoption behavior, this study addresses the first research gap and examines users' continuance usage behavior.

**Figure 1:**
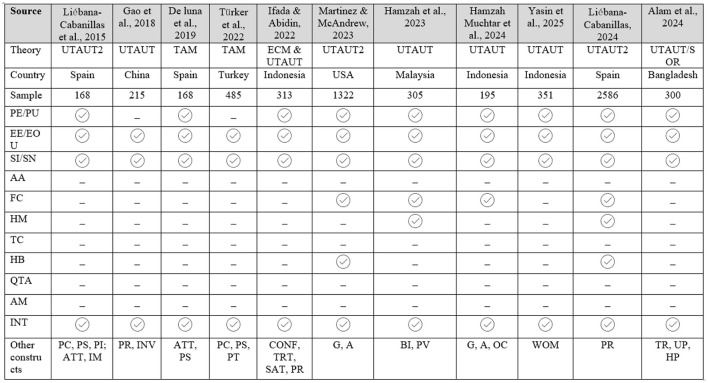
Summary of QR-code payment studies. CONF, Confirmation; TRT, Trust; SAT, Satisfaction; PR, Perceived risk; PE, Perceived expectancy; PU, Perceived usefulness; EE, Effort expectancy; EOU, Ease of use; SI, Social influence; SN, Subjective norm; AA, Alternative attractiveness; FC, Facilitating condition; HM, Hedonic motivation; AM, Amotivation; INT, Intention; TC, Transaction convenience; HB, Habit; ATT, Attitude; PS, Perceived security; INV, Involvement; BI, Brand image; PV, Perceived value; PI, Personal innovativeness; PC, Perceived compatibility; IM, Individual mobility; PT, Perceived trust; G, Gender; A, Age; OC, Occupation; TR, Trust; UP, Utilitarian Perception; HP, Hedonic perception; PB, Perceived benefit; WOM, Word of mouth.

Several researchers have examined users' intention and behaviors by employing multiple theories such as UTAUT, UTAUT2, TAM, TPB, etc (refer Figure 1). However, their studies were mostly conducted in developed countries context, which was unable to address least development country issues. Hence, this study employs a modified UTAUT2 model to measure the sustained usage intention of QR payments particularly in developing country context. By doing so, this study seeks to address the second research gap. Prior research on mobile payment adoption has predominantly emphasized facilitating factors, with limited attention given to inhibiting factors (refer Figure 1). This study seeks to address this imbalance by incorporating both enabling and constraining elements, thereby offering a more holistic understanding of the determinants of continuous usage intention and find the third research gap. Finally, this study also provides an novel insight of moderating role of AA and QTA, providing a new direction of post-adoption literature.

The aforementioned gaps contribute to answering the following research questions: (a) Among the main antecedents, which is the most influential that affects the QR-code mobile payment continuance intention? (b) Is the modified UTAUT2 model sufficient in explaining both enhancing and deterrent factors? (c) Does QR transaction anxiety moderate the relationship between facilitating condition and QR-code mobile payment continuance intention? (d) Does transaction convenience moderate the relationship between performance expectancy and QR-code mobile payment continuance intention? (e) What practical recommendations can be derived from the analysis of IPMA to effectively enhance user retention in QR-code mobile payment systems? To answer these research questions (a, b, c, d), we employ SEM analysis using PLS 3.0. In addition, IPMA is used to answer our research question (e).

This study makes several important contributions to the field of mobile payment research. First, it advances QR payment research by focusing on post-adoption stage, addressing a key gap in existing literature. Second, this study extends the UTAUT2 model by introducing both enhancement and deterrent factors, offering a more holistic understanding of sustained use. Finally, this study offers novel insights into the moderating roles of AA and QTA, thereby introducing a new perspective to the post-adoption literature and extending the current understanding of user behavior in the mobile payment context. In conclusion, this study deepens the understanding of continued usage intentions toward QR payments and provides valuable insights for researchers, governments, and MFS providers in emerging countries and beyond.

## 2 Literature review

### Prior QR-code m-payment studies

Numerous innovations have been introduced in mobile payment services, including QR code technology, which utilizes a black-and-white grid pattern to capture static information and can be easily scanned by smartphone (Gao et al., 2018). The topic of QR-MP has been widely investigated in previous research, which has focused on identifying the factors that influence user behavior toward payment systems at different stages, such as pre-adoption (intention), actual use, and post-adoption.

Investigations carried out at pre-adoption stages have aimed to explain the determinants from a range of theoretical perspectives. For instance, Yasin et al. (2025) used the UTAUT model to examine the factors influencing consumers' intentions to adopt QRIS payment, with their findings emphasizing word of mouth as the most critical factor. (Türker et al. 2022) examined the extended TAM model with additional factors such as trust, security, and compatibility. Similarly, (Ngo et al. 2021) measured Vietnamese customers' intention to use QR-MP by employing the TAM model. The findings revealed that all TAM constructs are supported and subjective norm to intention through attitude have full mediation. Using the UTAUT model, (Haritha 2022) examined how customer trust mediated between UTAUT base constructs and intentions to use mobile payment in the Indian economy. (Himel et al. 2021) modified the TAM model to include barrier and trust as additional constructs in their mixed-mode research. Their study revealed valuable insights by interviewing respondents and found all their constructs influence the intention to use mobile payment. Similarly, (Lian and Li 2021) studied the determinants of QR-code pay continuance usage intention, using the UTAUT2 model with users' trust dimension. In another study, (Kaur et al. 2020) examined both the intention and intention to recommend, and they employed innovation resistance theory (IRT) by distinguishing functional barriers and psychological barriers. Moreover, (Gao et al. 2018) investigated mobile payment and revealed perceived expectancy and effort expectancy showed significant path results. Reviewing prior mobile payment research shows that post-adoption stage studies have been less studied compared to pre-adoption studies. Furthermore, studies on the post-adoption stage have found UTAUT2 to be the appropriate model to predict QR-MP continuous behavior (Widayat and Masudin 2023); (Migliore et al. 2022). This research extends the findings of these studies by modifying the UTAUT2 base model. Figure 1 shows summary of previous QR-code-payment-based studies.

### 2.1 UTAUT2 model

The UTAUT model was developed by (Venkatesh et al. 2003) following a thorough assessment of 8 leading models. The four main factors of this model are performance expectancy, effort expectancy, social influence, and facilitating conditions. Despite its immense accessibility, Venkatesh again extended the theory of the UTAUT model in 2012, namely UTAUT2 where this model was made in the context of individual user acceptance. Three additional factors, namely hedonic motivation, price value, and habit, are added into the UTAUT2 model. The UTAUT2 framework has demonstrated greater effectiveness in improving both behavioral intention and usage behavior. Numerous scholars have utilized both the UTAUT and UTAUT2 models in studies across diverse areas, such as internet banking (Yuen et al., 2015), m-banking (Mahfuz et al., 2016), mobile payment (Migliore et al., 2022), NFC payment (Liébana-Cabanillas et al., 2021), e-wallet (Gupta and Arora, 2020).

Although it is a widely used technology acceptance model, UTAUT2 proved to be inadequate in fully obtaining specific task environments (Zarco et al., 2024). (Martins et al. 2014) also suggested in their m-payment research to analyse the UTAUT2 base model with relevant factors to get better insights into future technology research. Moreover, many scholars have revisited the base model, by removing and adding constructs (Morosan and DeFranco, 2016; Lian and Li, 2021). For instance, (Morosan and DeFranco 2016) have modified UTAUT2 model by excluding price value and including some additional factors to predict NFC m-payment intention. Similarly, (Mustafa et al. 2022) added perceived value, curiosity, perceived functional and environmental awareness and excluded effort expectancy to their 5G adoption study.

The original UTAUT2 model was primarily conceptualized to explain user behavior at the initial adoption stage of technology usage. While it effectively explains how individuals adopt mobile financial technologies, it is less capable of explaining post-adoption behaviors. Therefore, relying solely on UTAUT2 to understand continuous usage in such financial service environments may lead to incomplete or insufficient explanatory power. In response to these limitations, and informed by prior empirical research, this study modifies the UTAUT2 framework by incorporating four additional constructs, such as transaction convenience, amotivation, alternative attractiveness, and QR transaction anxiety. In addition, we excluded price value from the base model. Finally, the justification for including the additional constructs and excluding price value from the modified model is presented below:

i) **Price value:** In this study, we have excluded price value due to the irrelevant connection with our study context. The questions of this construct such as “QR-Mobile payment is reasonably priced. QR-Mobile payment is a good value for the money. At the current price, QR-mobile payment provides a good value” which is only relevant if QR-code mobile payment requires any charge. However, for QR-mobile payment, there is no charge associated and the apps required to conduct the payment are totally free. Prior several studies have removed this construct for the same reason (Morosan and DeFranco, 2016; Gupta and Arora, 2020; Istijanto and Handoko, 2022; Ly et al., 2025; Shankar and Rishi, 2020).

ii) **Transaction convenience:** QR code M-pay is a contactless payment system that requires a mobile phone device with an internet connection and a few steps, such as app login, QR code scanning, inserting amount, and payment (Ifada and Abidin, 2022). Although transaction convenience is associated with quick shopping, easy payment, and easy returns (Shankar and Rishi, 2020), it is a crucial factor to be considered by users when making payments. Moreover, transaction convenience is the post-use experience that influences a user's future usage intention (Shankar and Rishi, 2020). Therefore, we have included this factor in our current model.

iii) **Amotivation:** According to Vallerand (Pelletier et al., 2001), self-determined factors should be examined along with amotivation. Similarly, (Li 2017) suggested that amotivation should not be ignored when understanding user adoption of smart technology. An individual's post-use amotivation can arise after a certain technology has been adopted, and consequently it causes discontinuation of the existing technology. Therefore, we believe that amotivation is an appropriate construct to be examined for this study since our study is going to measure users' continuous intention.

iv) **Alternative attractiveness:** When it comes to continuing with existing technology, users often consider the functional benefits of other alternatives (Foroughi et al., 2024). If they find it attractive, they may discontinue using the current technology (Karjaluoto et al., 2020). For decades, payment systems have been continuously upgraded to be more advanced, with MFS providers consistently adding utility features to enhance security and convenience. Therefore, “alternative attractiveness” can be a significant factor influencing users' decisions in selecting the best alternatives. Hence, it is essential to include this construct in the current study context.

v) **QR transaction anxiety:** QR transaction anxiety reflects users' fear or worry about making mistakes, security breaches, or transaction failures during QR mobile payments (Al Amin et al., 2023). Such anxiety can reduce confidence and negatively affecting users' intention to continue using the technology (Tsai et al., 2025). Unlike the enhancement factor, this construct is able to bring out the negative aspects of users while using QR payment. Therefore, it is justified as an important relevant psychological factor of continuous intention of using QR payment systems.

### 2.2 Theoretical foundations and formation of hypotheses

This study's framework is based on UTAUT2 model with additional relevant factors. Moreover, the factors are categorized by both enhancement and deterrent factors. Enhancement factors refer to the elements that reinforce users' motivation to keep using that technology over time (Sharma and Mishra, 2023) and known as enabled factors. While deterrent factors refer to the elements that reduce the likelihood of engaging in a particular behavior (Choi, 2022). In the context of technology continuous intention, deterrent factors are the barriers that negatively impact users' intention to continue with technology.

Performance expectancy refers to an individual's belief that a specific technology will improve their performance (Venkatesh et al., 2003). Users who believe that mobile payment systems will provide tangible benefits, such as faster transaction times and enhanced security, are more likely to express a continuous intention to use these systems (Utomo et al., 2021). Furthermore, when technological platform meets users' performance expectations, it enhances satisfaction, which leads to their continued use of the technology (Yan et al., 2021). Many empirical evidences revealed that performance expectancy (PE) has a strong effect on users' intention to use different technologies, such as biometric m-payment (Liébana-Cabanillas et al., 2024), QR mobile payment (Shunmugasundaram et al., 2025; Liébana-Cabanillas et al., 2015), mobile financial service (Himel et al., 2021), fintech (Haritha, 2022), biometric payment (Zarco et al., 2024). Therefore, it can be argued that the higher the perceived usefulness of a technology, the stronger the intention to maintain its usage of QR-MP. Hence, the subsequent hypothesis could be formulated:

**H1:** PE is positively associated with continuance intention to use QR-code m-payment.

Effort expectancy, considered the second most crucial factor in the UTAUT2 model, refers to the level of effort a person perceives as necessary to complete a task using mobile payment services such as QR-code payments (De Luna et al., 2019). Essentially, when individuals believe that technology is easy to navigate, they are more likely to engage with it and maintain their usage over time (Ong et al., 2023). Prior study by (Chike and Ogba 2023) has concluded that, if the payment process is perceived as requiring little effort, users will experience less frustration and cognitive overload, contributing to higher continuance intention. Recent research has highlighted the significance of the relationship between effort expectancy (EE) and users' continued intention to use a product through satisfaction (Yan et al., 2021). In conclusion, it can be said that the easier a product is to use, the more likely users are to continue using it. Thus, the following hypothesis is proposed for this study:

**H2:** EE is positively associated with continuance intention to use QR-code m-payment.

Social influence, also referred to as subjective norm, represents the impact of an individual's chosen social referents in encouraging a specific behavior (Xiong et al., 2019). Prospective users tend to value the opinions of these individuals as important insights and give considerable weight to their recommendations. According to the theory of social influence, the approval of significant others can aid individuals in adopting a particular technology (Isaac and Sherali, 2014). In this study, individuals may be influenced by various social groups, which may offer guidance on the benefits or risks of using QR-MP technology. The more individuals accept these suggestions, the more likely they are to choose to use QR-code m-payment. Numerous prior studies on technology adoption have demonstrated the significant impact of social influence on behavioral intention (Hien et al., 2025; Cobanoglu et al., 2015; de Sena Abrahão et al., 2016; Istijanto and Handoko, 2022). Thus, it can be concluded that social influence is a strong predictor of continuous usage intention for QR-code m-payment. Therefore, we propose:

**H3:** SI is positively associated with continuance intention to use QR-code m-payment.

Facilitating conditions refer to the degree to which individuals believe that the available resources and technological infrastructure support the successful implementation of the system (Venkatesh et al., 2003). FC create a supportive environment that reduces the effort required to use QR Pay, making it easier for users to adopt and continue using the technology (Lee and Kwon, 2011). In the context of mobile payment systems, FC can include aspects such as technical infrastructure, user support, and access to necessary resources such as smartphones and internet connectivity (Tomić et al. 2023). A well-designed interface that simplifies the process of scanning QR codes and completing transactions can significantly enhance user satisfaction, resulting in higher intention to continue using it (Xu et al., 2024). Moreover, empirical study by (Widayat and Masudin 2023) have shown that positive FC contributes to a favorable attitude toward QR code payments, which in turn affects users' continuous intention. Thus, the following hypothesis is built:

**H4:** FC is positively associated with continuance intention to use QR-code m-payment.

Habit refers to a behavior or action that an individual carries out automatically as a result of learning and long-term experience (Ahuja and Khazanchi, 2016). (Venkatesh et al. 2012) emphasized the importance of prior experience in shaping individuals' intrinsic beliefs, which in turn have an impact on their behaviors. Moreover, MFS users associate QR code payments with specific contexts, such as paying for groceries, food, or shopping. These contextual cues trigger the habit loop, making users more likely to default to QR code payments in those situations. According to (Permana 2020), habit emerges as the primary factor influencing individuals' inclination to persist in using mobile payment platforms. Similarly, (Kurniawan et al. 2025) posited that habit is a significant predictor of QRIS adoption intention. Therefore, we propose:

**H5:** HAB is positively associated with continuance intention to use QR-code m-payment.

Hedonic motivation signifies the user's enjoyment in using information technology and the aspiration to use it repeatedly (Venkatesh et al., 2012), such motivation is form of intrinsic motivation, in which individual is experiencing fun and enjoyment while using any technology (Lee et al., 2015). In the post adoption stage, MFS user's hedonic motivation can come from device playfulness and its unique features. Empirical evidence from past studies has affirmed the effect of hedonic motivation on the post-adoption phases of mobile payment (Ly et al., 2025; Sleiman et al., 2022; Lian and Li, 2021; Liébana-Cabanillas et al., 2021). Therefore, the use of QR-code mobile payment may enhance the entertainment and fascination experienced by MFS users during transactions, thereby leading to a positive impact on their mobile payment service continuous use. Thus, we propose:

**H6:** HM is positively associated with continuance intention to use QR-code m-payment.

Transaction convenience refers to the “speed and ease with which consumers can complete the transaction” (Benoit et al., 2017). Reducing the time and effort needed to complete transactions is crucial for transactional convenience (Jiang et al., 2013). The preference of consumers for using a payment method is influenced by how convenient they find it for making payments (Boden et al., 2020). Transaction convenience is a significant factor motivating consumers to transition to online platforms, where transactions can be rapidly completed through ease scanning techniques (Shankar and Rishi, 2020). The greater the convenience user experiences during transactions, the more likely users are to continue using the platform (Lian and Li, 2021). Moreover, TC facilitates easier interactions, leading to increased satisfaction and repeat usage of technology (Hossain et al., 2024). Although it is evident in prior studies that PE influences technology continuous intention (Dayour et al., 2020), the inclusion of TC as moderator could influence the existing relationship. For example, users may get operational effectiveness from QR-pay, but lower transaction convenience could discourage them from continuing to use the system regularly. In contrast, higher transaction convenience can increase user's satisfaction and loyalty (Sui and Geng, 2021). Thus, the hypothesis below is developed:

**H7:** TC is positively associated with continuance intention to use QR-code m-payment.

**H7a:** TC moderates the relationship between performance expectancy and continuance intention to use QR-code m-payment.

According to cognitive social theory, anxiety can be categorized into two types: state anxiety and trait anxiety (Spielberger and Smith, 1966). State anxiety refers to the emotional state of anxiety typically experienced prior to and during an event, while trait anxiety is associated with the inherent personality traits and reflects a more general tendency to experience anxiety (Saadé and Kira, 2007). In the context of this study, users encounter state anxiety during QR-code m-payment transactions, which arises from situational factors such as concerns about internet disconnection, low battery levels, entering incorrect payment details, or recalling payment authentication codes (Lin and Hsieh, 2023). These temporary stressors can negatively affect users' continuous intention to use QR-MP. Although facilitating conditions generally enhance users' continuance intention by providing essential resources, guidance, and support (Hien et al., 2025), however, transaction anxiety may weaken this positive effect. Because when anxiety is high, users feel unsure and nervous during digital payments, which lowers their confidence and makes them less likely to benefit from the available facilitating conditions. In such cases, even good facilitating conditions may not lead to continuance intention because anxiety makes users focus more on risks and possible mistakes than on support and convenience. Conversely, when transaction anxiety is low, users are more likely to rely on and benefit from facilitating conditions, thereby strengthening their intention to continue using QR-code m-payment. Empirically, prior research has shown that anxiety can moderate individuals' online transaction intentions (Ferreira da Silva et al., 2024; Jeng et al., 2022), supporting the potential moderating role of transaction anxiety in technology adoption contexts. Therefore, we propose the following hypothesis:

**H8:** QTA is negatively associated with continuance intention to use QR-code m-payment.

**H8a:** QTA moderates the relationship between facilitating condition and continuance intention to use QR-code m-payment.

Alternative attractiveness refers to the evaluation of the potential fulfillment that can be obtained from different goods or services (Liao et al., 2021). Due to the growing competition in the digital financial market, MFS and banks are continuously launching new MFS products to establish the fastest and safest payment system. In this context, the alternative attractiveness comes from other payment platforms, like cash, bank debit/credit card, NFC-enabled bank card, SMS-based pay, and wearable payment device, etc. When users perceive that other payment options are more attractive and convenient, they are more likely to discontinue the existing payment platform. The study conducted by (Loh et al. 2021) established the influence of alternative attractiveness on the behavioral intention of users to use mobile payment applications. Similarly, (Foroughi et al. 2024) have stated that attractiveness of other travel apps reduces customer's continuous intention. Therefore, the hypothesis is proposed:

**H9:** AA is negatively associated with continuance intention to use QR-code m-payment.

Amotivation is characterized by an individual experiencing adverse outcomes while engaging in activities, leading to feelings of anxiety, distraction, disengagement, and overall negative consequences (Pelletier et al., 2001). According to (Ryan and Deci 2000), amotivation represents the lowest level of self-determination as it signifies a state where individuals experience a complete lack of personal control and intrinsic motivation. Furthermore, amotivation should not be seen as a direct contrast to intrinsic and extrinsic motivations. Rather, it is more accurate to consider these concepts as existing on a continuum, with varying levels of self-determination impacting behavior, ranging from high to low (Deci and Ryan, 1985). Amotivation arises at two stages: pre-use and post-use. At the pre-use stage, technology's advantages and disadvantages are not known to individuals, thus preventing them from forming an opinion about its use, which in turn affects their motivation to adopt it (Kaikkonen, 2019). In the post-use stage, the individual is familiar with technology, has experienced its benefits, and can form an opinion about it. Prior studies have found a negative relation in many. For instance, a very recent study by (Stiegemeier et al. 2024) examined drivers' intention to use in-vehicle technology and found amotivation a significant factor. Similarly, (Donaldson and Duggan 2014) found an inverse correlation between amotivation and continuance intention to use information systems. In current study context, QR-code m-pay users experiences post-usage amotivation, and we assume that this will lead to a negative correlation with its continuous intention. Thus, the hypothesis is built for this study:

**H10:** AM is negatively associated with continuance intention to use QR-code m-payment.

## 3 Research method

### 3.1 The measures

This study uses a 7-point Likert scale (1 = “strongly disagree,” 7 = “strongly agree”) to measure all of its constructs. Table 1 depicts the construct's adapted sources, study context and CR value. The table also shows all constructs are reflective in nature. As the original instrument for the variables in this model was formulated in English, current research employs a back-translation approach to translate from English to Bengali for the purpose of developing the preliminary Bengali questionnaire. To ensure the face and content validity of the questionnaire, three mobile financial service experts and two academicians conducted a review to confirm that the content was valid, clear, meaningful, and easily understood. Based on the experts' recommendations, several changes were brought into the final questionnaire. Subsequently, 35 MFS users were invited to participate in pilot-testing. Based on satisfactory CR and AVE results from the pilot test, the final questionnaires were subsequently distributed to the respondents.

**Table 1:** Instrumentation source.

**Variable**	**Adapted source**	**Study context**	**Construct nature**	**Number of items**
Performance expectancy	de Sena Abrahão et al., 2016	Mobile payment	Reflective (0.89)	Three
Effort expectancy	Liébana-Cabanillas et al., 2021	NFC payment	Reflective (0.836)	Three
Transaction convenience	Shankar and Rishi, 2020	Mobile banking	Reflective (0.787)	Three
Social influence	Lian and Li, 2021	Mobile payment	Reflective (0.923)	Three
Alternative attractiveness	Foroughi et al., 2024	Travel apps	Reflective (0.957)	Four
Facilitating condition	Istijanto and Handoko, 2022	Mobile payment	Reflective (0.898)	Four
Amotivation	Rosli and Saleh, 2023	E-wallet	Reflective (0.828)	Three
Habit	Gupta and Arora, 2020	Mobile payment	Reflective (0.828)	Four
Hedonic motivation	Hamzah et al., 2023	QR-code M-payment	Reflective (0.836)	Three
QR transaction anxiety	Lin et al., 2023	Online transaction	Reflective (0.929)	Four
Continuous intention	Yan et al., 2021	Health apps	Reflective (0.847)	Four

### 3.2 Data collection and sampling

As suggested by (Merhi et al. 2019), online data collection is more effective and is of high quality than offline collection. Therefore, online data were collected from August 2024 to October 2024 by sharing survey links to multiple social media groups such as bKash (MFS-5.1M members), Daraz (m-commerce, 16M members), and Bikroy (Buy and sell group-4M). Since we are measuring continuous usage intention of QR code payment, it is essential to collect data from relevant participants. This study employed purposive sampling, including participants who had adopted QR-pay but used it infrequently. Infrequent use was defined as using QR-pay less than once per week or not at all in the past month. Eligibility was assessed through a brief screening questionnaire that asked whether participants had ever used QR-pay, how often they currently used it, and when they last used it. Only participants whose self-reported answers met the criteria were invited to complete the full questionnaire. This method ensured that all participants met the intended inclusion criteria while maintaining transparency and allowing the study to be replicable. The sample size for our study was determined using G-power analysis due to the lack of a complete sample frame. The study requires a minimum sample size of 184 participants, calculated using G-power analysis with an effect size of 0.15, a significance level (α) of 0.05, a power (1–(β)) of 0.95, and 12 predictors. Finally, 247 usable questionnaires were accumulated, with 46% response rate.

### 3.3 Common method variance (CMV)

To minimize the potential impact of common method bias (CMB), this study applied both procedural and statistical techniques, as recommended by (Podsakoff et al. 2024). For procedural remedies, we implemented several strategies during the design and administration of the survey. These included (a) ensuring respondent anonymity and confidentiality, and (b) using clear and concise language in the survey to avoid ambiguity. In addition, for the statistical procedures, three tests were conducted. First, the analysis of Harman's single-factor test shows that the first factor accounted for 23.53% of the total variance, well below the 50% threshold, indicating that common method variance (CMV) is unlikely to be a major concern (Podsakoff et al., 2024). Second, a complete collinearity test was performed to assess multicollinearity among all constructs. As shown in Table 2, no variance inflation factor (VIF) values exceeded the thresholds of 10 (Kalnins and Praitis Hill, 2025), confirming that multicollinearity was not an issue in the dataset. Finally, the marker variable technique (Rönkkö and Ylitalo, 2011) was employed and found no significant changes in the results, with only a minor 3.27% adjustment in the (*R*^2^) value (see Table 3) for the dependent variable (CINT), confirming that common method variance is not a significant issue. The summarization of all CMB method analysis results is depicted in Table 4.

**Table 2:** Full collinearity test with VIF values.

**Variables**	**Inner VIF**
Alternative attractiveness	1.141
Performance expectancy	2.253
Amotivation	1.318
Effort expectancy	2.386
Continuance intentions	1.623
Transaction convenience	1.223
Facilitating condition	1.472
Habit	1.513
Hedonic motivation	1.149
Social influence	1.277
QR transaction anxiety	1.441

**Table 3:** Marker variable result.

**Target variable**	**Without marker variable**	**With marker variable**	**Change in *R*^2^**
Continuous intention	0.764	0.789	3.27%

**Table 4:** CMB analysis summary.

**CMB analysis**	**Standard threshold**	**Our findings**	**Remarks**
Harman's one-factor test	< 50% variance Acceptable (Podsakoff et al., 2024)	23.53	Acceptable
Full collinearity test	VIF >10 Strong indication of CMB (Kalnins and Praitis Hill, 2025)	1.149–1.623	No CMB issue
Marker variable test	Δ*R*^2^>5% Potential CMB issue (Rönkkö and Ylitalo, 2011)	3.27% *R*^2^ change	Minor issue

### 3.4 Data analysis

In this study, PLS-SEM is applied to examine the direct influence of deterrent and enhancement factors on the continued intention to use QR-code mobile payments, along with assessing the moderating effects. PLS-SEM is particularly suitable for this analysis due to its ability to model complex relationships, including direct, indirect, and moderating effects, while accommodating small sample sizes and non-normal data distributions (Hair et al., 2012). Furthermore, importance-performance map analysis (IPMA) is performed to offer actionable insights for managerial implications, helping identify the factors that should be prioritized to improve user continuance intention.

## 4 Results

### 4.1 Descriptive results

Descriptive statistics were employed to analyze the demographic samples, with the participants' demographic characteristics and are presented in Table 5. It represents that all respondents were aged 18 and above. Men comprised 45.3% of respondents, and women comprised 54.6%, while students accounted for 14.9%, service holder 49.7%, business and housewives who were QR-code m-payment users accounted for 23% and 10.5%, respectively. For internet packages, 47.9% subscribed to 7-day packs, whereas 37.1% prefer 30 days and 9.3% users subscribed to unlimited packages. The descriptive analysis also shows that 60% and 30.2% respondents use only mobile wallet apps and mobile banking apps, whereas 9.9% are using both. Finally, the statistical findings indicate that 44.5% of users were banked and 55.4% were unbanked.

**Table 5:** Demographic representation.

**Measure**	**Items**	**Frequency**	**Percent**
Gender	a) Male	91	36.7
	b) Female	157	63.3
Age	a) 18–25	103	41.5
	b) 26–33	125	50.4
	c) 34–41	17	6.8
	d) 42 <	3	1.2
Types of MFS	a) Mobile wallet apps	149	60
	b) Mobile banking apps	75	30.2
	c) Both	24	9.9
Frequency of MFS use (per month)	a) 1-3 times	52	21
	b) 4–10 times	118	47.6
	c) 11–20 times	75	30.2
	d) 21 <	3	1.2
Occupation	a) Student	137	55.2
	b) Service holder-Private/Public	72	29.2
	c) Business	23	9.2
	d) Housewives	12	4.8
	e) Others	4	1.6
Educational level	a) Secondary School	1	0.4
	b) Higher Secondary school	53	21.3
	c) Under graduation	114	45.9
	d) Bachelor	52	20.9
	e) Master	25	10.3
	f) Ph.D.	3	1.2
Banking access	a) Banked	110	44.5
	b) Unbanked	137	55.4
Type of internet packs	a) 7 days	119	47.9
	b) 30 days	92	37.1
	c) Unlimited	23	9.3
	d) Never buy	14	5.7

### 4.2 Outer model analysis

Table 6 provides an evaluation of the constructs' reliability and validity. Cronbach's Alpha (CA) was employed to examine reliability in this study, and all values were greater than 0.7, indicating that the variables are reliable. To assess internal consistency reliability, Dijkstra–Henseler's rho (rho-A) and composite reliability (CR) were used, with a threshold of 0.7. The results demonstrated that all variables had CR values greater than 0.7, confirming the high reliability of the items. Besides that, the results showed that every item had AVE values ranging from 0.582 to 0.736, which above the stated minimal criterion of 0.50.

**Table 6:** Outer model summary.

**Variables**	**Loadings**	**α**	**CR**	**AVE**
Alternative attractiveness (AA)	0.843	0.859	0.861	0.674
	0.838			
	0.795			
	0.808			
QR transaction anxiety (QTA)	0.869	0.880	0.918	0.736
	0.827			
	0.831			
	0.902			
Amotivation (AM)	0.764	0.771	0.866	0.684
	0.868			
	0.845			
Effort expectancy (EE)	0.800	0.816	0.878	0.644
	0.821			
	0.792			
	0.796			
Facilitating condition (FC)	0.840	0.787	0.875	0.701
	0.843			
	0.829			
Habit (HA)	0.845	0.759	0.861	0.674
	0.808			
	0.808			
Hedonic motivation (HM)	0.759	0.817	0.885	0.722
	0.865			
	0.917			
Performance expectancy (PE)	0.830	0.847	0.897	0.686
	0.814			
	0.855			
	0.814			
Social influence (SI)	0.891	0.819	0.889	0.729
	0.910			
	0.753			
Transaction convenience (TC)	0.719	0.786	0.846	0.582
	0.884			
	0.799			
	0.724			
Continuance intentions (CINT)	0.831	0.849	0.898	0.688
	0.818			
	0.816			
	0.853			

Discriminant validity is crucial for assessing whether measurements of distinct components, which may have some overlap, are indeed separate and not overly correlated (Ramayah et al., 2017). To address this, the research employed methods such as the loadings (refer to Table 7), and Fornell–Larcker criterion (Table 7). The conceptual model is considered to demonstrate significant discriminant validity, since all correlations are depicted as being below the square root of the average variance extracted (AVE) (Saadé and Kira, 2007).

**Table 7:** Analysis of discriminant validity.

**Constructs**	**AA**	**AM**	**CINT**	**EE**	**FC**	**HA**	**HM**	**PE**	**QTA**	**SI**	**TC**
Fornell-Larcker criterion											
AA	0.689										
AM	0.029	0.827									
CINT	–0.152	–0.508	0.830								
EE	–0.049	–0.383	0.724	0.802							
FC	–0.069	–0.222	0.575	0.509	0.837						
HAB	–0.063	–0.246	0.576	0.387	0.310	0.821					
HM	0.214	0.060	-0.091	–0.134	–0.102	–0.080	0.850				
PE	–0.154	–0.365	0.725	0.647	0.476	0.522	–0.089	0.828			
QTA	0.111	0.385	–0.539	–0.421	–0.286	–0.355	0.168	–0.410	0.858		
SI	0.179	–0.176	0.275	0.246	0.137	0.281	0.209	0.217	–0.122	0.854	
TC	–0.032	–0.129	0.199	–0.116	0.060	0.184	0.094	0.113	–0.122	0.189	0.763

SI, Social influence; AA, Alternative attractiveness; FC, Facilitating condition; HM, Hedonic motivation; AM, Amotivation; CINT, Continuance Intention; EE, Effort expectancy; PE, Performance expectancy; TC, Transaction convenience; HB, Habit.

### 4.3 Inner model analysis

To test the hypothesized relationships, we employed bootstrapping with 10,000 resamples, as guided by recent research (Hair et al., 2012). Based on the SEM-PLS analysis, inner model evaluation results (see Table 8) revealed that performance expectancy (H1), effort expectancy (H2), facilitating condition (H4), and habit (H5) significantly affected MFS user's QR-MP continuance intention (CINT) (respectively, β = 0.324, *t* = 3.838; β = 0.310, *t* = 7.027; β = 0.169, *t* = 4.599). Moreover, the four additional constructs–transaction convenience (H7, β = 0.117, *t* = 3.432); QR transaction anxiety (H8, β = −0.117, *t* = 3.962); alternative attractiveness (H9, β = −0.080, *t* = 2.278); and amotivation (H10, β = −0.172, *t* = 5.072)–were also found to be supported.

**Table 8:** Inner model analysis results.

**H**	**Relation**	**β**	***t***	***p***	**PCI-LL**	**PCI-UL**	**f^2^**	**S/Ns**	**R^2^**
H1	PE → CINT	0.192	3.838	0.000	0.108	0.271	0.078	S	0.764
H2	EE → CINT	0.310	7.027	0.000	0.242	0.387	0.192	S	
H3	SI → CINT	0.009	0.275	0.391	–0.045	0.058	0.000	Ns	
H4	FC → CINT	0.169	4.599	0.000	0.110	0.232	0.092	S	
H5	HAB → CINT	0.172	4.797	0.000	0.116	0.233	0.093	S	
H6	HM → CINT	0.040	1.321	0.093	–0.001	0.097	0.007	Ns	
H7	TC → CINT	0.117	3.432	0.000	0.061	0.174	0.054	S	
H8	QTA → CINT	–0.117	3.962	0.000	–0.167	–0.071	0.046	S	
H9	AA → CINT	–0.080	2.278	0.011	–0.131	–0.016	0.027	S	
H10	AM → CINT	–0.172	5.702	0.000	–0.223	–0.124	0.107	S	

SI, Social influence; AA, Alternative attractiveness; FC, Facilitating condition; HM, Hedonic motivation; AM, Amotivation; EE, Effort expectancy; CINT, Continuance Intention; PE, Performance expectancy; TC, Transaction convenience; QTA, QR transaction anxiety; PCI-LL, Percentile Confidence Interval-Lower Limit; PCI-UL, Percentile Confidence Interval-Upper Limit; S, Supported; Ns, Not supported; HB, Habit; ns, not supported; β, standardized regression weights; *p*, probability; Bootstrapping based on *n* = 10,000 bootstrap samples (***p < 0.001, **p < 0.01, *p < 0.05).

**Figure 2:**
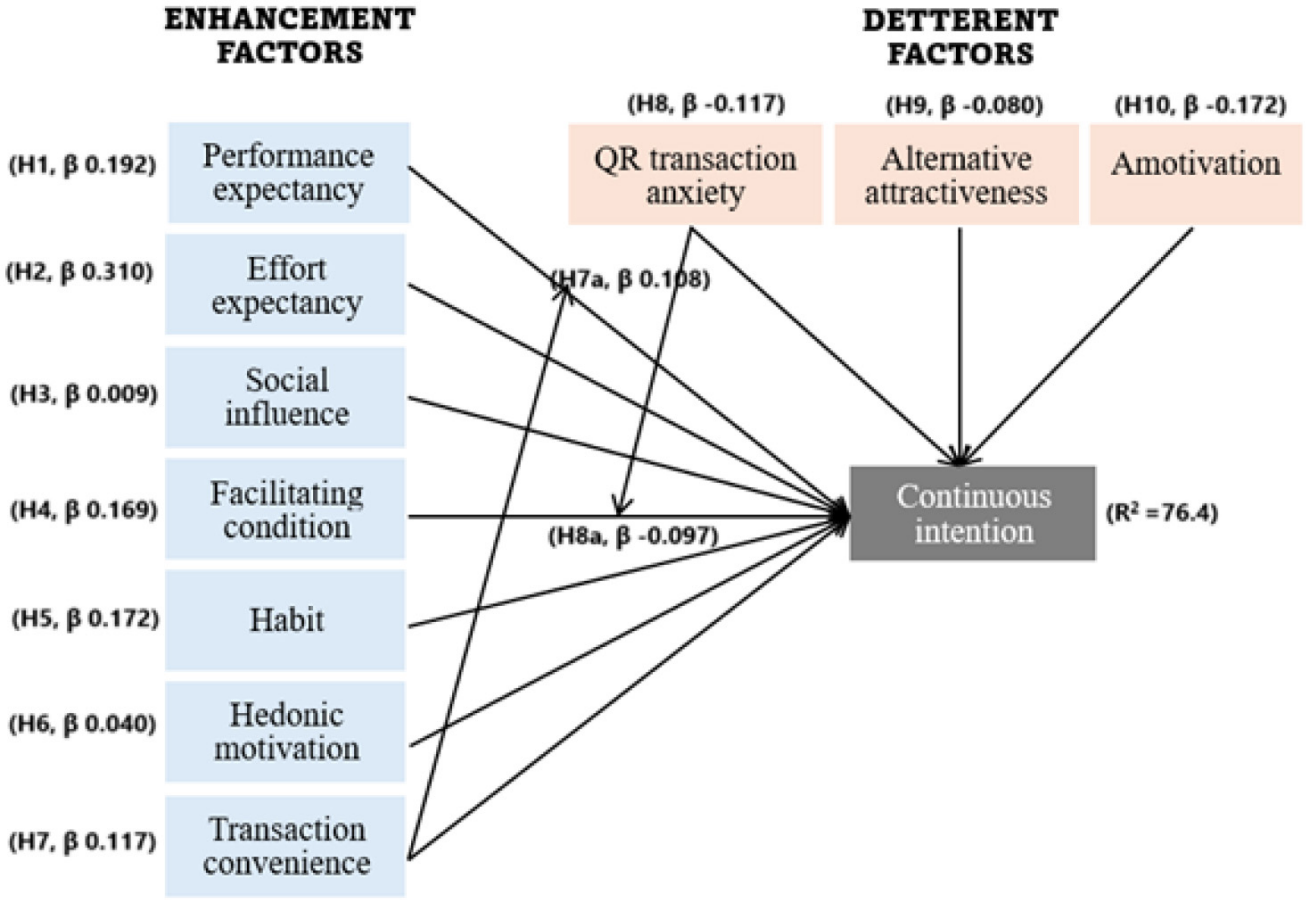
Research framework.

However, social influence (H3) and hedonic motivation (H6) were not statistically significant (β = 0.009 and β = 0.040, respectively) predictors of continuance intention. The empirical model explained 76.4% of the variance in continuance intention (see Figure 2). The high *R*^2^ reflects strong model fit, confirming that the predictors explain substantial variance and support the theoretical model. Although high explained variance may sometimes raise concerns about overfitting, the use of theoretically grounded constructs and acceptable multicollinearity diagnostics (VIF values) provides confidence that the model's explanatory power is not artificially inflated.

Furthermore, effect sizes (*f*^2^) were evaluated according to Cohen's (1988) guidelines, which classify *f*^2^ values of 0.02, 0.15, and 0.35 as small, medium, and large effects, respectively. The results indicate that SI and HM exert no significant effect, while PE, FC, QTA, AM, HA, TC, and AA demonstrate a small effect, and EE has a medium effect on the target variable CINT (see Table 7).

### 4.4 Moderation analysis

The current study explored the moderating role of QR transaction anxiety (QTA) on the relationship between facilitating conditions (FC) and QR-code mobile payment continuous intention (CINT). In the absence of the moderating effect, the R^2^ value for CINT was 0.793, indicating that FC explained 76.4% of the variance in CINT. However, when the interaction term was included, the R^2^ value increased to 0.79, reflecting a 3.27% increase in the variance explained in the target variable, CINT. Additionally, the significance of the moderating effect was examined, and the results revealed a negative and significant moderating impact of QTA on the relationship between FC and CINT (β = –0.097, *t* = 2.319, *P* = 0.010), supporting H11. This suggests that as QR transaction anxiety increases, the relationship between FC and CINT weakens. Furthermore, another moderating effect, TC, was assessed, and the results showed a positive and significant moderating impact of TC on the relationship between performance expectancy (PE) and CINT (β = 0.108, *t* = 3.051, *P* = 0.001), supporting H12. The summary is depicted in Table 9.


$$f^{2}=\frac{R_{\text{included moderator}}^{2}-R_{\text{excluded moderator}}^{2}}{1-R_{\text{included moderator}}^{2}}$$

$$f^{2}=\frac{0.790-0.764}{1-0.790}=0.123$$

**Table 9:** Moderation results.

**H**	**Relationship**	**β**	**t**	**R^2^**	**P**	***f* ^2^**	**Supported**
H7a	TC*PE → CINT	0.108	3.051	0.79	0.001	0.040	YES
H8a	QTA*FC → CINT	–0.097	2.319	–	0.010	0.037	YES

**Figure 3:**
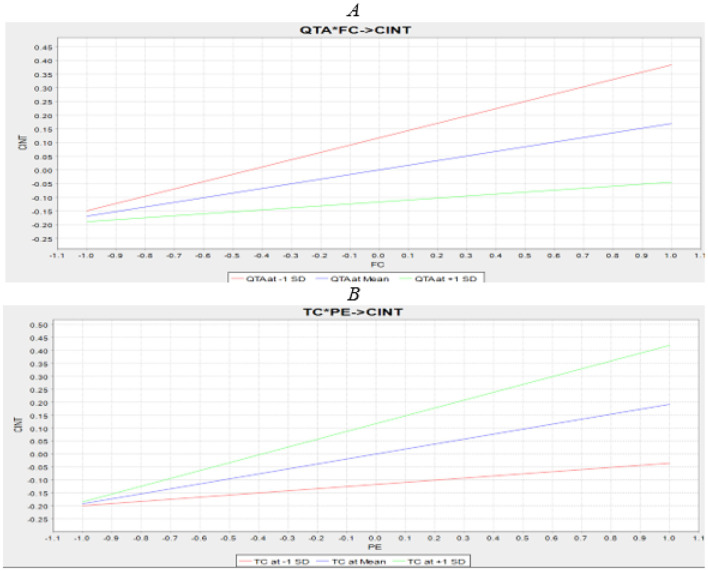
**(A, B)** Moderation effect slop analysis.

In this study, we adhere to the recommendations of (Zarco et al. 2024) regarding effect sizes, as they provide more dependable values. With an effect size of 0.123, it can be inferred that the effect size is large (Shunmugasundaram et al., 2025). The slope lines represent the effect of FC on CINT at three levels of QTA: one standard deviation below the mean (low QTA, red line), at the mean (average QTA, blue line), and one standard deviation above the mean (high QTA, green line). Additionally, as shown in Figure 3A, the line representing high QTA has a steeper slope compared to the line for low QTA, suggesting that the positive relationship is stronger when QTA is high. Therefore, it can be concluded that the influence of facilitating conditions (FC) on continued intention (CINT) is weaker with higher QTA. Similarly, Figure 3B illustrates the moderation relationship for TC and improves the positive relationship between PE and CINT.

### 4.5 The importance-performance map analysis (IPMA)

**Figure 4:**
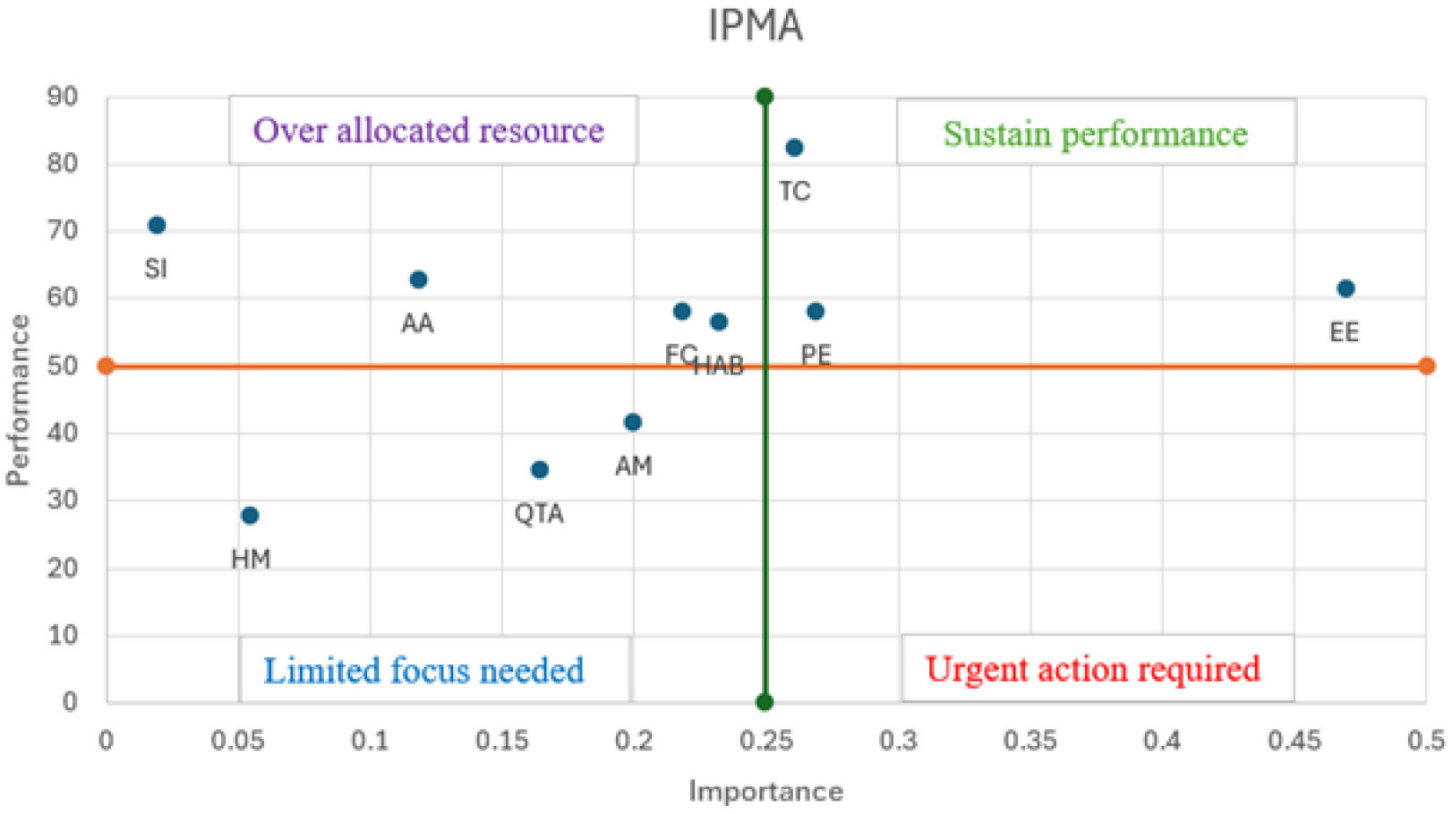
IPMA scatter plot.

According to (Ringle and Sarstedt 2016), “The IPMA gives researchers the opportunity to enrich their PLS-SEM analysis and, thereby, gain additional results and findings. More specifically, instead of only analysing the path coefficients (i.e., the importance dimension), the IPMA also considers the average value of the latent variables and their indicators (i.e., performance dimension).” IPMA results are presented by the two-dimensional graph (see Figure 4), where the horizontal axis describes the “importance,” and the vertical axis describes their performance. The 50% cut-off used to distinguish high vs. low performance and importance in the IPMA analysis is based on the conventional midpoint rule commonly applied in IPMA and managerial decision-making research. Since IPMA performance values in Smart-PLS are rescaled to a 0–100 metric, the midpoint (50) represents a natural theoretical boundary separating low from acceptable performance levels (Ringle and Sarstedt, 2016). The graphs in Figure 3 and Table 10 reveal that the most important construct was EE, followed by PE, TC, HAB, FC, AM, QTA, AA, and SI. The result of the IPMA provides valuable insights for MFS providers, enabling them to locate both high/low-performing factors and facilitates the development of immediate strategies aimed at retaining current customers while also attracting potential new ones.

**Table 10:** The importance and performance values of constructs are derived from the IPMA analysis.

**Factors**	**Importance**	**Performance**	**Category**
Alternative attractiveness	0.12	62.66	High performance, low importance
Amotivation	0.20	41.49	High importance, low performance
Effort expectancy	0.47	61.30	High importance, high performance
Facilitating condition	0.22	58.01	High performance, low importance
Habit	0.23	56.44	High performance, low importance
Hedonic motivation	0.06	27.77	Low importance, low performance
Performance expectancy	0.27	58.02	High importance, high performance
QR transaction anxiety	0.16	34.44	High importance, low performance
Social influence	0.02	70.75	High performance, low importance
Transaction convenience	0.26	82.39	High importance, high performance

## 5 Discussion

### 5.1 Relationship between performance expectancy and CINT

Our study supports the correlation between performance expectancy (PE) and continuous intention, showing that MFS users derive utilitarian benefits from using QR-code payments. Such as quick processing (Nguyen, 2024), totally contactless and speed up checkout process (Ong et al., 2023). Due to the ease feature of it, users find it more useful and convenient for their daily payment. Besides, for non-banked MFS users, a QR-code is very useful since they don't have access to traditional banking facilities. Our results are in line with the findings of several prior studies, such as mobile food delivery apps (Munday and Humbani, 2024); social mobile apps continuously using intention (Akdim et al., 2022); Mobile payment (Lian and Li 2021); QR-MP (Gao et al., 2018); 5G usage intention (Mustafa et al., 2022).

### 5.2 Relationship between effort expectancy and CINT

The results revealed that EE is supported positively and also aligned with other studies (Migliore et al., 2022; Esawe, 2022; Sonia et al., 2024). In general, modern apps are continuously upgraded with advanced features, which MFS providers are continuously developed based on users' demand. For instance, the MFS app log-in system, which was initially designed to insert a pin code every time a user logs into their account. However, the process has been upgraded with biometric log-in, which requires no extra effort. That means users' continuous intention toward any technology depends on its continuous development of user-friendly features.

### 5.3 Relationship between social influence and CINT

Another non-supported construct in this study shows SI. Prior empirical studies have consistently revealed its lack of correlation with intention. For instance, (Nguyen 2024) determined that, social influence is deemed less relevant in the context of individuals making decisions about whether to continue using banking applications. In a similar vein, (Cheng et al., 2020) found that users' continued intention to use mobile news apps is influenced by factors such as perceived usefulness, hedonic motivation, habit, and facilitating conditions. However, our findings contradicted with (Ifada and Abidin 2022), where they highlighted that the intention to continue utilizing QR code m-payment services is affected by the recommendations of individuals in one's social network, such as family members, friends, relatives, or coworkers. In our study, social influence may not be derived from the following sources. Perhaps, users may encourage to continue QR payment due to the personal preferences or functional benefits of the system. Finally, for the non-supported result we may say that social influence appears to be more relevant before adoption, while it becomes less influential after users have adopted it.

### 5.4 Relationship between facilitating condition and CINT

This study's empirical findings highlight the significant role of facilitating conditions (FC) in shaping the continued intention (CINT) to use QR-code mobile payments. These results are consistent with the recent study by (Martinez and McAndrews 2023). Additionally, essential facilities for QR-MP, such as fast internet connections, reliable Wi-Fi, Android or iOS smartphones, high-resolution QR-code stickers, dual-language application support, and biometric login options, are identified as key factors for ensuring fast and convenient transactions. Ultimately, if MFS users are provided with the necessary technological and other related facilities, they are more likely to continue making payments via QR-MP.

### 5.5 Relationship between habit and CINT

This study revealed the positive effect of HAB on CINT to use QR-MP, which is similar to the prior mobile payment studies (Martinez and McAndrews, 2023; Sleiman et al., 2022; Karjaluoto et al., 2020; Lin and Hsieh, 2023). In Bangladesh, a large number of retail shops with a QR-enable payment system, facilitating consumers to purchase their daily commodities with QR-MP, become a payment norm. Furthermore, the emergence of super apps that integrate various payment gateways into a single platform has encouraged users to rely on a single device for all their payment needs and establish a dependency on QR-MP.

### 5.6 Relationship between hedonic motivation and CINT

The path analysis results of the current study revealed that hedonic motivation (HM) is not supported, a finding that aligns with previous mobile payment studies (Wu et al., 2021; Sleiman et al., 2022). The non-supported results may arise because MFS users may initially find this QR-code payment as fun or enjoyment because of its “Scan to Pay” feature; however, for continuous usage of it, they may use it for their utilitarian benefits rather than enjoyment. Besides, users may be attracted by the external reward offered by MFS providers such as “buy one get one free, cash back, and point per transaction.” This may undermine the users' hedonic motivation. Therefore, in this study, they don't find any relevance with hedonic motivation to continuous usage of the QR payment system.

### 5.7 Relationship between transaction convenience and CINT

Our statistical results empirically proved the correlation between TC and CINT. The outcome is aligned with previous research by (Shankar and Rishi 2020). Practically, users found the transaction process through QR-payment is more prominent than other payment platform like debit/credit card. QR-payment doesn't require any POS terminals or additional devices, which makes this payment faster and more convenient. Moreover, QR-payment reduces the queuing time while paying bills. This study also found TC significantly moderate relationship between PE and CINT. For instance, when TC is high, users experience the full benefits of the system's performance (i.e., speed, efficiency, and ease of use). If users feel the transaction process is seamless and requires minimal effort, their perceived satisfaction with the system's performance increases, thereafter, strengthen the link between performance expectancy and their intention to continue using the service.

### 5.8 Relationship between QR transaction anxiety and CINT

One of the deterrent factors, QR transaction anxiety, has been identified as a significant predictor of continuous intention (CINT), with a negative impact on its prediction. The prior study by (Jeng et al. 2022) also shows similar findings. Furthermore, the moderating effect of QTA also revealed that it weakens the relationship between FC and the CINT to use QR-code mobile payments. This may reflect that even if users perceive the necessary resources FC to be in place, their anxiety about potential disruptions or challenges during the transaction can lead to hesitation or reluctance to continue using QR payments.

### 5.9 Relationship between alternative attractiveness and CINT

Furthermore, AA also have found supported and have an inverse relationship. This negative correlation is supported may be based on the following reasons. For example, there are two types of MFS users: individuals have bank accounts and individuals don't have bank accounts. For bank account holders, they have multiple payment options (Such as debit/credit card, NFC enable card, wearable device) rather than cash. For them, they may get additional benefits from using “Tap N Pay” NFC-oriented debit/credit card, considered as one of the fastest and most secured payment platforms. Besides, it doesn't require users to carry any device or internet connection. In addition, even if the purchases are made with the wrong amount, the transaction can be reverted easily. Such benefits may attract MFS users to discontinue the existing payment platform. However, for the unbanked MFS users, since they don't have any bank account, thereby their alternative payment option is only cash. For them, to continue QR-code payment, it is often required to cash in or top up cash to their MFS mobile apps for payment. As a result, the need for frequent cash top-ups lowers the relative advantage of QR-code payments since they can easily make direct cash payments instead.

### 5.10 Relationship between amotivation and CINT

The results also found a significant negative correlation between AM and continuous intention. Prior studies have shown similar results by (Rosli and Saleh 2023). In our study context, users are experienced post-use amotivation because they may think the psychological disadvantages of this payment mode is higher than its advantages. There might be a number of reasons. At first, when users adopted this QR-pay mode, they were not aware of the future consequences. However, while continuing, they may face many difficulties or discomforts. For example, using QR-code payments or less transparent payment methods reduces the pain of paying (Dash et al., 2023; Prelec and Loewenstein, 1998), which can increase compulsive buying or small, frequent purchases (Seldal and Nyhus, 2022). This can negatively affect users' financial wellbeing, leading to feelings of guilt, shame, or inadequacy (Huang et al., 2024). Consequently, users may be amotivated toward such type of platforms as QR-payment. In addition, post-adoption challenges, such as mobile internet connectivity and a series of authentication procedures for each transaction, can impose a psychological burden on users, potentially arises amotivation toward the usage of QR-code mobile payments. To overcome these issues, MFS provider can upgrade their payment network infrastructure, such as an offline payment system (without internet) to eliminate the all-time mobile internet connectivity issue. In addition, the number of authentications needs to be reduced and included more advanced log-in features like voice recognition and face detection mechanisms. Such upgrading features can help users to reduce amotivation and understand the relative advantages of the existing payment system.

## 6 Conclusion

This study develops a theoretical framework, augmenting the IS continuance model by distinguishing all UTAUT2 (except price value) constructs along with newly added constructs–transaction convenience as enhancement factors and AA, QTA, and AM as deterrent factors to explain CI to use QR-code m-payment. Based on the findings at this stage, our study successfully addresses the stated research questions–RQ(a) EE is the most influential factor influential that affect the QR-code mobile payment continuance intention. RQ(b) except for the non-significant effect of SN and HM, most hypotheses from enhanced UTAUT2 dual factor model are supported. RQ (c & d) this study confirms the positive moderating effect of TC on the relationship between PE and users' CINT, as well as the negative moderating effect of QTA between FC and users' CINT. RQ(e) the IPMA analysis identified the prioritized factors and provide actionable recommendations for enhancing user retention.

### 6.1 Theoretical contribution

This study offers several key theoretical contributions to the field of mobile payment research. First, by focusing on the post-adoption stages of QR-code mobile payment systems, this research addresses a significant gap in the existing literature, which has predominantly concentrated on pre-adoption behaviors and user intentions. Exploring post-adoption behaviors provides deeper insights into the factors that sustain or hinder continued use, an area critical for the long-term success of mobile payment platforms. Second, this study extends the UTAUT2 model by introducing both enhancement and deterrent factors, thus broadening the understanding of users' sustained engagement with QR-code mobile payments. The integration of these new constructs offers a more comprehensive framework for analysing the drivers and barriers influencing post-adoption behavior. Additionally, by examining moderating effect through the lens of deterrent factors (QTA) and enhancement factor (TC), this research provides a clear understanding of how different influences may either intensify or lessen users continued use of mobile payment technologies.

### 6.2 Managerial implication

In an emerging economy country, mobile wallet businesses are very competitive. Therefore, retaining the market share become a great challenge for MFS business owners. The findings of this study will assist MFS providers in developing business strategies and improving user experience. This study offers several recommendations derived from the outcomes of the IPMA. For example, EE is the most important and high-performing construct influencing continued usage. This suggests that Bangladeshi users are more likely to keep using QR payment services when the system remains simple, convenient, and easy to operate. Service providers such bKash should focus on maintaining a simple UX design and minimize steps during transactions. Besides they can enable Bengali voice support for each feature. Similarly, PE also strongly affects continuance intention. In Bangladesh, users are likely to continue using QR mobile payments when they perceive tangible and practical benefits from the service. MFS providers can enhance this by offering features such as digital receipts for government or utility payments and automatic reminders for recurring bills. In addition to that, integration with local merchants and microfinance services can increase the everyday utility of QR payments. TC also falls into this same category, suggested to ensure that the app works across multiple platforms and devices. To further enhance convenience, MFS providers could integrate biometric authentication systems, allowing users to complete payments securely without going through multiple verification steps. Additionally, factors such as FC, AA, and HAB, while exhibiting strong performance, hold relatively lower importance and are, therefore, considered overemphasized. As a result, MFS providers can optimize resource allocation by shifting focus toward more critical service areas. Since facilitating conditions are performing well but lower important, MFS providers can reduce the frequency of updates to support systems without compromising service quality. Also, they can focus on maintaining efficient technical support to address customer needs only when needed. Similarly, for alternative attractiveness, marketers should reduce efforts aimed at competing with other financial products and focus on improving core services that have a direct impact on customer loyalty and satisfaction. Moreover, expending excessive resources to further encourage habitual behavior may lead to diminishing returns, as it may have only a limited impact on user engagement. Besides, over-investment in habit formation could lead to neglect of other areas that might drive new customer acquisition or expand service usage. Therefore, MFS provider could focus on expanding the ecosystem of businesses that accept QR code payments, or integrating value-added features such as loyalty programs, rewards, or cashback incentives. These features can enhance customer satisfaction and retention without solely relying on habitual behavior. Finally, factors such as QTA and AM, which are of low priority according to the IPMA findings, require minimal attention. For instance, financial service providers should optimize the QR payment process by providing intuitive, error-free interfaces and ensuring a reliable, seamless internet connection for smooth transaction execution.

### 6.3 Limitations and future recommendations

Although we made considerable efforts to examine all relevant aspects of the topic, but our study has some limitations.

- This study employed a cross-sectional survey, which may not fully capture users' post-adoption behavior over time. Therefore, it is suggested that future studies adopt a longitudinal approach to observe changes in behavior at various time intervals.
- This study discusses solely on QR-code payment, although different types of mobile payment are associated with different behavioral patterns (Liébana-Cabanillas et al., 2021). Moreover, with the growing variety of cashless transaction system (sound waves-based payments, NFC payment, magnetic secure transmission payments, etc), it would be valuable to see how different mobile payment platform affect users' continuous intention.
- Current study constructs are based on individual and apps context; future studies can examine government regulation on QR-payment behavior.
- This study collected data from social media groups which may have introduced sampling bias, as these users are typically more digitally active than the broader population. Future studies should adopt more diverse sampling methods.
- This study focused solely on moderating variables and did not explore any mediating constructs. Consequently, future research should consider the inclusion of mediating variables such as satisfaction and trust.
- In the current study, hedonic motivation was found to be a non-significant construct. Future research could explore the possible crowding-out effect to gain deeper understanding and potentially reveal new theoretical contributions.
- This study is specifically focused its key dependent variable as “continuance intention to use.” Future research can investigate other post-adoption behaviors, such as switching behavior and loyalty.
- Finally, while the current study focuses on a least-developed economy, future research could conduct a cross-economic study between developing and developed economies to gain broader insights.

## Data Availability

The raw data supporting the conclusions of this article will be made available by the authors, without undue reservation.
